# The business of radiology

**DOI:** 10.4103/0971-3026.73526

**Published:** 2010-11

**Authors:** Bhavin Jankharia

**Affiliations:** Editor-in-Chief, The Indian Journal of Radiology and Imaging “F”, 1^st^ Floor, Bhaveshwar Vihar, 383, Sardar V P Road, Mumbai – 400 004, India. E-mail: editor@ijri.org

Last month, I was with a young radiology MD student who was escorting me to the venue of a conference that I was speaking at. He studies in a private medical college. The fee he had paid for entry into the radiology MD course was Rs. 1.0 crore, i.e., Rs. 10 million and the current going rate is Rs. 15 million. I was told that most other private medical colleges that offer MD courses in radiology in the country have similar fee structures, as well.

This article is not about the rights/wrongs and ethics of this practice, which is now clearly widespread across the country. I want to concentrate, in the piece, on the misconceptions about incomes in radiology.

I asked the resident just one question. If he had known at the time of admission, what he knew now as a second year resident, would he still have paid the money? His answer was a clear “No”.

The reason a student pays this kind of money to enter radiology is related to one of two situations. The student may be the offspring of a radiologist who perhaps wants him/her to take over an existing practice. If the son/daughter does not get an MD radiology seat through the entrance examinations, the parent may be willing to pay a high price for this seat, assuming it to be just another investment for the future. This article is not about them.

I am focusing on the second group, i.e., those, like the resident who was accompanying me, who truly believe before admission that radiology and radiologists make tons of money as compared to other disciplines. This belief probably comes from the outward perception that CT scans and MRI machines generate large revenues, which in turn has led to a misconception among the lay public as well as non-radiology medical professionals, that there is a disproportionate amount of income to be made in radiology as compared to other professions.

This is so untrue. The capital expenditure required to start CT scan and MRI centers is huge. The return on investment at best hovers around 12–15%, which is a return that can even be obtained through market investments in debt funds and index equity funds. Competition is fierce and most centers earn just enough to pay back their loans. It is only the depreciation that allows a radiology entrepreneur/businessman to generate some income. Also, as far as salaried radiologists are concerned, individual radiologists at best can hope to make between Rs. 1 and 6 lakhs per month, depending on their experience and expertise.

This is essentially on par with other disciplines in medicine, when we compare the average 80%. There will always be a 20% of the doctor population that will earn disproportionately due to a variety of factors. For example, a cardiac surgeon who performs five surgeries per day will earn an astronomical amount of money, which is not really the norm for cardiac surgeons throughout the country. Similarly, some radiologists may be able to pull in disproportionate income due to a combination of unusual factors that are usually very difficult to replicate, e.g., great terms with a successful hospital that has outsourced its radiology, or presence in a completely untapped market, or an exceptionally successful interventional practice, etc.

Recovering the Rs. 10 or 15 million investment in an MD radiology seat is not easy. And, it may be a good idea for the public at large and medical students in particular to understand these facts.

Many radiologists do not understand the business aspects of the profession they work in. However, sooner or later, a working knowledge of basic issues related to accounting, processes, human resources, etc. becomes a must, irrespective of whether the radiologist is in private practice, an entrepreneur or working in a hospital or even in the government. Given the large capital expenditures involved, this knowledge or lack of it can make or break a radiology department.

It may not be a bad idea to introduce “Business of Radiology” as a subject during radiology residency. It may also not be a bad idea to introduce “Business of Medicine” as a subject during MBBS days. A business run ethically can enhance a medical practice significantly and there is no point going through life with blinders simply because we have been led to believe over the years that the practice of medicine and “business” cannot be talked about in the same breath. This is not true, especially in radiology.

Strong, reasoned opinions on this subject will be published as Letters to the Editor in the February issue, if received by December 15.

